# Prevalence, Risk Factors, and Impact of Undiagnosed Visually Significant Cataract: The Singapore Epidemiology of Eye Diseases Study

**DOI:** 10.1371/journal.pone.0170804

**Published:** 2017-01-27

**Authors:** Jacqueline Chua, Blanche Lim, Eva K. Fenwick, Alfred Tau Liang Gan, Ava Grace Tan, Ecosse Lamoureux, Paul Mitchell, Jie Jin Wang, Tien Yin Wong, Ching-Yu Cheng

**Affiliations:** 1 Singapore Eye Research Institute, Singapore National Eye Centre, Singapore, Singapore; 2 Duke-NUS Medical School, Singapore, Singapore; 3 Centre for Eye Research Australia, Royal Victorian Eye and Ear Hospital, University of Melbourne, Melbourne, Victoria, Australia; 4 Centre for Vision Research, Department of Ophthalmology and Westmead Institute for Medical Research, University of Sydney, Sydney, New South Wales, Australia; 5 Department of Ophthalmology, Yong Loo Lin School of Medicine, National University of Singapore, Singapore, Singapore; Soochow University Medical College, CHINA

## Abstract

**Objective:**

To determine the prevalence, risk factors, and impact of undiagnosed visually significant cataract in an Asian population.

**Methods:**

The Singapore Epidemiology of Eye Diseases is a population-based study where 8,697 adults of Malay, Indian, and Chinese ethnicities aged > 40 years were invited for an eye examination, including lens photograph, to establish cataract diagnosis. Visually significant cataract was defined by Wisconsin Cataract Grading System and a best-corrected visual acuity <20/40 with cataract as the primary cause of vision impairment. Participants were deemed ‘undiagnosed’ if they had visually significant cataract and reported no prior physician diagnosis of cataract. Visual functioning (VF) was assessed with the VF-11 questionnaire validated using Rasch analysis.

**Results:**

Among the 925 participants with visually significant cataract, 636 (68.8%) were unaware of their cataract status. Age-standardized prevalence varied according to ethnicity, with Malays having higher rates than Chinese and Indians. Factors independently associated with having undiagnosed visually significant cataract were: Malay ethnicity, lower educational attainment, in employment, and without a history of diabetes (all P<0.05). In those with undiagnosed visually significant cataract, half had bilateral visual impairment, which was significantly associated with 24.8% poorer visual functioning compared to those with unilateral visual impairment (P<0.001).

**Conclusions:**

Two-thirds of Singaporean adults with visually significant cataract were previously undiagnosed. Half of these cases had bilateral visual impairment and substantially reduced quality of life. Public health strategies targeting elderly patients, such as regular screening for visual impairment and timely referral to ophthalmologists in order to prevent progression to bilateral visual impairment when visual function is compromised are warranted.

## Introduction

Cataract is the leading cause of blindness and second cause of visual impairment worldwide, affecting 46 million people.[1, 2] Despite being readily treatable, cataract continues to be the major cause of visual impairment among elderly adults in developed countries, including Singapore.[3, 4] As the population continues to age rapidly, the number of people experiencing vision loss as a result of cataract is anticipated to grow. Individuals with visually significant cataract tend to experience symptoms such as blurred vision, loss of contrast, halos, difficulty with glare, which can considerably affect patients’ ability to perform day-to-day tasks.[5] Studies have also shown increased mortality in older persons with cataract, after accounting for known mortality risk factors.[6, 7] However, vision loss from cataract can be effectively restored with modern cataract surgery, which is considered among the most cost-effective interventions in health care[8] leading to substantially improved quality of life in most patients.[9]

While population-based studies have explored the prevalence of cataract/cataract surgery in Asia,[10–18] the literature on rates of undiagnosed cataract is limited. It is plausible that patients may adapt to their visual impairment and may fail to notice functional decline as a result of undiagnosed cataract. Understanding the prevalence, risk factors and visual functioning associated with undiagnosed visually significant cataract may inform public health strategies to increase eye health awareness and access to eye screening services with the potential to reduce the burden of visual impairment from undiagnosed cataract from both the patient and societal perspective.

Using data from the Singapore Epidemiology of Eye Diseases (SEED) Study, a population-based, cross-sectional study of eye disease in adult Asians (Chinese, Malays and Indians) living in Singapore, we aimed to determine the prevalence, risk factors, and impact of undiagnosed visually significant cataract. We hypothesized that the prevalence of undiagnosed visually significant cataract would be high but would vary according to ethnicity and socio-demographic factors, and that those with undiagnosed visually significant cataract would have poor visual functioning.

## Methods

### Study population

The Singapore Epidemiology of Eye Diseases (SEED) Study comprises of 3 population-based studies on the 3 major ethnic groups in Singapore: Malays (2004–2006),[19] Indians (2007–2009),[20] and Chinese (2009–2011).[20] Details of the study design and methodology have been described previously.[19, 20] Briefly, an age-stratified (by 10-year age groups) random sampling in each ethnic group was used to select 4,168 Malays, 4,497 Indians and 4,606 Chinese aged 40 and above. Of these, 3,280 Malays, 3,400 Indians and 3,353 Chinese participated in the study. The overall response rate was 75.6%. Study participants were older than non-participants (*P*<0.001), but there was no gender difference (*P* = 0.68). Written Informed consent was obtained from all participants. Approval for conducting this study, this study including the consent procedure, was obtained from the Singapore Eye Research Institute Institutional Review Board, and all study procedures adhered to recommendations of the Declaration of Helsinki. Procedures related to the present study are presented below.

### Examination procedures

Each participant underwent a comprehensive, standardized eye examination. Presenting and best-corrected visual acuity (VA) were measured using a logarithm of the minimum angle of resolution (Log MAR) number chart (Lighthouse International, New York, USA) at 4 meters, for each eye. Presenting VA was obtained with the participants wearing their ‘‘walk-in” optical correction (spectacles or contact lenses), if any. Best-corrected VA was measured with best possible correction obtained with subjective refraction.

Lens opacity was assessed using the Wisconsin Cataract Grading System.[21, 22] After dilation, lens photographs were taken by digital slit-lamp (Topcon model DC-1 with FD-21 flash attachment; Topcon, Tokyo, Japan) and retroillumination (Nidek EAS-1000; Nidek, Japan) cameras. All photographs were graded by a single experienced grader (AGT) at the University of Sydney who also graded cataract for the Blue Mountains Eye Study.[22] Inter- and intra-grader reliabilities for the three types of cataract were high, as reported previously.[23] A person with visually significant cataract was defined by having, in either eye: Wisconsin grading of either nuclear (opacity of ≥grade 4), cortical (opacity of ≥25% of the lens) or posterior subcapsular (PSC) cataract (opacity of ≥5% of the lens), and a best-corrected VA worse than Log MAR 0.30 (20/40) in the cataractous eye, with cataract as the primary cause of vision impairment.[24] Cataract surgery was defined as the presence of an intraocular lens implant (pseudophakia) or the absence of the crystalline lens (aphakia), recorded during lens assessment during slit lamp examination at the study visit.[25] We defined participants as having bilateral visual impairment, defined as best-corrected VA worse than Log MAR 0.30 (20/40) in both eyes and unilateral visual impairment, defined as best-corrected VA worse than Log MAR 0.30 (20/40) in one eye and better than Log MAR 0.30 (20/40) in the other.

### Questionnaire

A standardized questionnaire was administered in English, Chinese, Malay or Tamil, depending on participants’ preferences, by trained interviewers prior to the ocular examination.[19, 20] Participants were asked, “Have you ever been told by a doctor that you have cataract?”. Their responses were coded as ‘‘Yes,” ‘‘No,” ‘‘Don’t know,” and ‘‘Unobtainable.” To ensure the accuracy of the responses of these subjects, our trained interviewers ensured that the cataract status referred to the *unoperated* eye. For all participants who answered “yes” to the question: “Have you ever been told by a doctor that you have cataract”, an additional follow-up question was asked to identify the eye that has cataract. Moreover, in all participants, we will ascertain their cataract surgical status via the following question: ‘‘Have you had an operation for cataract (i.e. right or left eye)?” The cataract surgical status was further corroborated with slit-lamp biomicroscopy findings. Participants with visually significant cataract were deemed ‘undiagnosed’ if he/she responded ‘‘no” to the question whereas were deemed ‘diagnosed’ if he/she responded ‘‘yes” to the question. Other questionnaire data included age, gender, ethnicity (Malay/Indian vs. Chinese),[26] housing type (small; 1- to 2-room public housing vs. large; 3 room public housing or bigger, including private housing), living arrangement (living alone vs. living with others), marital status (never married/ divorced/separated/ widowed vs. married), education (≤6 years of education vs. >6 years), employment status (retired/not working vs. working), individual monthly income (<$2000 vs. ≥$2000) and smoking status (current/past smoker vs. never smoker). Participants were asked if they used any form of visual aids and those who answered yes were then asked how often they visited any optometrists, opticians or ophthalmologists to check their eyes and for glasses/contact lens. In addition, participants were asked if they were aware of any deterioration of their general vision.

### Other measurements

Seated blood pressure (BP) and blood samples were collected during the examinations. Hypertension was defined as systolic BP ≥140mmHg, diastolic BP ≥90mmHg, physician diagnosed hypertension or self-reported history of hypertension. Diabetes mellitus was defined as random glucose of ≥11.1 mmol/L, diabetic medication usage or a physician diagnosed history of diabetes. Hyperlipidemia was defined as total cholesterol ≥6.2mmol/L or self-reported use of lipid lowering drugs. Comorbidities included: self-reported angina, self-reported heart attack, self-reported stroke, self-reported thyroid condition, hyperlipidaemia and hypertension and were categorized into presence of ≥2 comorbidities vs. <2 comorbidities.

### Visual functioning

The Visual Function Index (VF)-11 questionnaire was used to assess the impact of cataract on participants’ visual functioning.[27] The VF-11 is a modified version of the VF-14 that has been culturally adapted for Singapore.[27] It comprises 11 questions which assess the level of difficulty associated with performing vision-related activities, such as reading and cooking. Rasch analysis, a form of item response theory, was used to assess the psychometric properties of the VF-11 in the current study sample, and subsequently to convert the raw VF-11 scores into interval-level Rasch person measures, expressed in log of odds units or logits. We used the overall VF Rasch-transformed score in our analysis.

### Statistical analyses

The normality of the data was assessed with the Shapiro–Wilk test. To compare the characteristics of participants among groups, the Kruskal-Wallis test was performed for non-normally continuous variables and chi-square tests for categorical variables. The prevalence estimate and its 95% confidence interval (CI) of undiagnosed visually significant cataract were calculated and standardized to the population distribution from the 2010 Singapore Census.[26] Briefly, the “number of persons at risk for undiagnosed visually significant cataract” was defined as the number of persons in each age group, excluding those (1) with bilateral pseudophakia/aphakia, (2) if both eyes had missing data, or (3) either eye had missing data and the other eye did not have visually significant cataract. To determine factors associated with undiagnosed visually significant cataract, multivariable logistic regression models were performed while adjusting for potential confounders. In addition to age, gender, and ethnicity, factors with P < 0.10 in the age-gender-ethnicity model were included in the multivariable model. The association between laterality of visual impairment (independent variable) and vision-specific functioning score (dependent variable) was assessed by multivariable linear regression models while adjusting for potential confounders. Data were analyzed using Stata 12.1 (Texas, USA).

## Results

**Fig 1:**
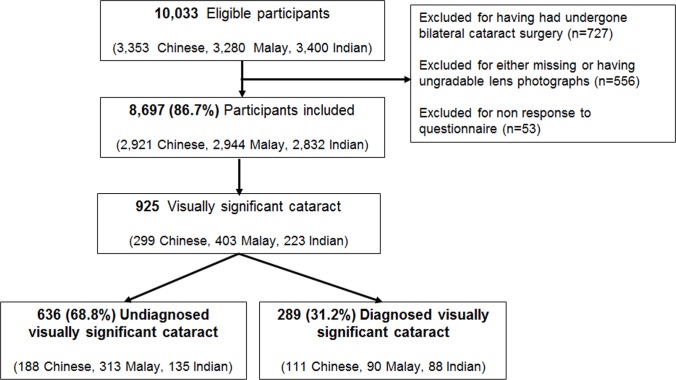
Flow chart of study participants. Among those with visually significant cataract, 68.8% (n = 636) had previously undiagnosed cataract. Unknown responses were not reported.

Of the 10,033 participants aged 40 years and above, bilateral cataract surgery had been performed in 727 participants, leaving 9,306 participants (3,086 Chinese, 3,125 Malay and 3,095 Indian) whose lens was present in one or both eyes (**Fig 1**). Of these, 8,697 (93.5%) participants had gradable lens photographs as well as complete questionnaire data. Participants excluded as a result of ungradable photographs and/or missing questionnaire data (See **S1 Table**) were more likely to be older, of Indian ethnicity, live in a smaller housing type, be retired or not working, have lower income, have diabetes, hypertension, hyperlipidemia, and have more comorbidities (*P*<0.05). Among the 8,697 participants included in the analysis, 925 (10.6%) had visually significant cataract in either eye.[24] Among those with visually significant cataract, 636 (68.8%) were unaware of their cataract status and were deemed as having undiagnosed visually significant cataract (**Fig 1**). In terms of ethnicity, Malays were more likely to report being unaware of their cataract status than Chinese and Indians (77.7% vs. 62.9% and 60.5%; both *P*<0.001).

### Prevalence of undiagnosed visually significant cataract

After age-standardization to the Singapore population of 40 years or older (**Table 1**), the prevalence of undiagnosed visually significant cataract was 6.54%; 95% confidence interval [CI], 5.88–7.30%, where it was highest among Malays (8.10%; 95%CI, 6.44–10.36%) and similar between Chinese (5.71%; 95%CI, 4.79–6.82%) and Indians (5.54%; 95%CI, 4.39–7.05%). Overall, women had significantly higher age-standardized prevalence of any undiagnosed visually significant cataract compared to men (7.88% vs. 5.35%; *P*<0.001). Such gender disparity was seen for all ethnic subgroups. The overall age-standardized prevalence of undiagnosed visually significant cataract was considerably higher (19.02%; 95%CI, 16.95–21.39%) among subjects aged 60 years or older.

**Table 1:** Prevalence of Undiagnosed Visually Significant Cataract Stratified by Age, Gender and Ethnicity.

Age groups	All Ethnic Groups		Chinese		Malay		Indian	
(years)	n	%	n	%	n	%	n	%
40–49	12	0.51	3	0.44	2	0.26	7	0.78
50–59	43	1.47	11	1.04	16	1.79	16	1.65
60–69	174	8.10	52	6.82	68	10.04	54	7.63
70–79	76	31.39	106	27.60	220	37.87	50	21.46
> 80	31	38.75	16	36.36	7	53.85	8	34.78
**Crude rate**	636	7.31	188	6.44	313	10.63	135	4.77
Standardized rate ≥40 years old (95%) *	6.54 (5.88, 7.30)		5.71 (4.79, 6.82)		8.10 (6.44, 10.36)		5.54 (4.39, 7.05)	
Standardized rate ≥60 years old (95%) *	19.02 (16.95, 21.39)		16.85 (13.99, 20.32)		24.03 (18.78, 31.27)		15.28 (11.73, 20.03)	
	**Male**	**Female**	**Male**	**Female**	**Male**	**Female**	**Male**	**Female**
**Crude**	6.58	8.03	6.25	6.62	9.28	11.87	4.25	5.30
Standardized rate ≥40 years old (95%) *	5.35 (4.55, 6.33)	7.88 (6.81, 9.15)	4.86 (3.70, 6.47)	6.62 (5.23, 8.36)	6.25 (4.31, 9.42)	9.99 (7.34, 14.02)	4.70 (4.31, 6.52)	6.85 (4.54, 10.66)
***P* value for between gender comparison**	<0.001		0.042		<0.001		0.012	

Abbreviations: CI = confidence interval.

* Age-standardized rates (95% CI) compared with the 2010 Singapore population.

### Sociodemographic and clinical characteristics of participants with undiagnosed vs. diagnosed visually significant cataract

The mean age of the 925 participants with visually significant cataract was 70.48 ± 7.74 years and 55.4% were female. Participants with undiagnosed visually significant cataract were similar in age (*P* = 0.530) and gender (*P* = 0.779) to those with diagnosed visually significant cataract (**Table 2**). Compared to those with diagnosed cataract (n = 289), persons with undiagnosed cataract were more likely to be of Malay ethnicity, live in a smaller housing type, live alone, have ≤6 years of education, and be without a history of diabetes. 50.5% (n = 321) of the undiagnosed cases were bilaterally visual impaired due to cataract (**Table 2**).

**Table 2:** Characteristics of Participants with Visually Significant Cataract Stratified by Undiagnosed vs. Diagnosed.

Characteristics	Total	Undiagnosed	Diagnosed	*P* value *
	(n = 925)	(n = 636)	(n = 289)	
Age, years	70.48 (7.74)	70.96 (7.32)	70.58 (7.72)	0.530
**Age groups**				
40–59 Years	86 (9.30)	55 (8.65)	31 (10.73)	0.313
≥60 Years	839 (90.70)	581 (91.35)	258 (89.27)	
**Gender**				
Male	413 (44.65)	282 (44.34)	131 (45.33)	0.779
Female	512 (55.35)	354 (55.66)	158 (54.67)	
**Ethnicity**				
Chinese	299 (32.32)	188 (29.56)	111 (38.41)	<0.001
Malay	403 (43.57)	313 (49.91)	90 (31.14)	
Indian	223 (24.11)	135 (21.23)	88 (30.45)	
**Housing type**				
Large	782 (84.63)	522 (82.08)	260 (90.28)	0.001
Small	142 (15.37)	114 (17.92)	28 (9.72)	
**Living arrangement**				
Living with others	859 (93.07)	582 (91.51)	277 (96.52)	0.006
Living alone	64 (6.93)	54 (8.49)	10 (3.48)	
**Marital status**				
Married	573 (61.95)	393 (61.79)	180 (62.28)	0.887
Never married, separated, divorced, widowed	352 (38.05)	243 (38.21)	109 (37.72)	
**Education**				
> 6 years	101 (10.94)	55 (8.65)	46 (16.03)	0.001
≤ 6 years	822 (89.06)	581 (91.35)	241 (83.97)	
**Employment status**				
Employed	190 (20.54)	137 (21.54)	53 (18.34)	0.052
Retired	341 (36.86)	218 (34.28)	123 (42.56)	
Not working	394 (42.59)	281 (44.18)	113 (39.10)	
**Monthly income**				
≥ $2000	28 (3.08)	19 (3.03)	9 (3.18)	0.904
< $2000	882 (96.92)	608 (96.97)	274 (96.82)	
**Diabetes**				
No	595 (66.85)	431 (70.66)	164 (58.57)	<0.001
Yes	295 (33.15)	179 (29.34)	116 (41.43)	
**Hypertension**				
No	150 (16.22)	104 (16.35)	46 (15.92)	0.868
Yes	775 (83.78)	532 (83.65)	243 (84.08)	
**Hyperlipidaemia**				
No	398 (44.42)	275 (44.43)	123 (44.40)	0.995
Yes	498 (55.58)	344 (55.57)	154 (55.60)	
**Comorbidities**				
<2	395 (44.58)	279 (45.66)	116 (42.18)	0.335
≥2	491 (55.42)	332 (54.34)	159 (57.82)	
**Smoking status**				
Never smoked	608 (65.87)	417 (64.83)	197 (68.17)	0.090
Ex-smoker	203 (21.99)	136 (21.45)	67 (23.18)	
Current smoker	112 (12.13)	87 (13.72)	25 (8.65)	
**Eyecare utilization pattern**				
Wears glasses of any kind				
Yes	604 (65.37)	409 (64.41)	195 (67.47)	0.364
No	320 (34.63)	226 (35.59)	94 (32.53)	
Yearly glasses checks				
Yes	90 (14.90)	55 (13.45)	35 (17.95)	0.146
No	514 (85.10)	354 (86.55)	160 (82.05)	
**Aware of vision deterioration**				
Yes	558 (60.65)	370 (58.54)	188 (65.28)	0.053
No	362 (39.35)	262 (41.46)	100 (34.72)	
**Ocular characteristics**				
**Laterality of visual impairment**				
Unilateral visual impairment	513 (55.46)	315 (49.53)	198 (68.51)	<0.001
Bilateral visual impairment	412 (44.54)	321 (50.47)	91 (31.49)	
**Previous cataract surgery**				
Yes	171 (18.61)	0 (0.00)	171 (59.58)	<0.001
No	748 (81.39)	632 (100.00)	116 (40.42)	
**Vision-specific functioning score**	2.76 (1.49)	2.82 (1.43)	2.63 (1.61)	0.181

Data presented are means (standard deviations) or number (%), as appropriate for variable.

Abbreviations: logMAR = logarithm of the minimum angle of resolution; VA = visual acuity.

* P value was obtained with Kruskal-Wallis for non-normally distributed continuous variables and chi-square for categorical variables.

### Factors associated with undiagnosed visually significant cataract

After adjusting for age, gender, ethnicity, and other significant factors found in the initial model, Malay ethnicity (odds ratio [OR], 1.98; 95%CI, 1.35–2.90; *P*<0.001), ≤6 years of education (OR, 1.67, 95%CI, 1.03–2.70; *P* = 0.037), in employment (OR, 2.08, 95%CI, 1.30–3.33; *P* = 0.002), without a history of diabetes (OR, 1.80, 95%CI, 1.31–2.50; *P*<0.001), bilateral visual impairment (OR, 2.56, 95%CI, 1.81–3.63; *P*<0.001), and better visual functioning scores (OR, 1.26, 95%CI, 1.12–1.42; *P*<0.001) were associated with higher odds of having undiagnosed visually significant cataract (**Table 3**).

**Table 3:** Factors Associated with Undiagnosed Visually Significant Cataract.

Characteristics	Model 1*		Model 2†	
	OR (95% CI)	*P* value	OR (95% CI)	*P* value
Age, per 10 years	1.01 (0.84, 1.22)	0.908	1.00 (0.79, 1.27)	0.985
**Age groups**				
40–59 Years	Reference		NA	
≥60 Years	1.15 (0.72, 1.86)	0.559	NA	NA
**Gender**				
Male	Reference		Reference	
Female	1.02 (0.77, 1.36)	0.881	1.05 (0.68, 1.63)	0.819
**Ethnicity**				
Chinese	Reference		Reference	
Malay	2.05 (1.47, 2.86)	<0.001	1.98 (1.35, 2.90)	<0.001
Indian	0.91 (0.63, 1.30)	0.603	1.00 (0.65, 1.53)	0.997
**Housing type**				
Large	Reference		Reference	
Small	1.63 (1.03, 2.56)	0.037	1.34 (0.80, 2.24)	0.263
**Living arrangement**				
Living with others	Reference		Reference	
Living alone	2.55 (1.27, 5.13)	0.009	1.83 (0.86, 3.90)	0.118
**Marital status**				
Married	Reference		NA	
Never married, separated, divorced, widowed	0.93 (0.67, 1.28)	0.645	NA	NA
**Education**				
> 6 years	Reference		NA	
≤ 6 years	1.72 (1.10, 2.70)	0.018	1.67 (1.03, 2.70)	0.037
**Employment status**				
Retired	Reference		Reference	
Employed	1.97 (1.27, 3.05)	0.003	2.08 (1.30, 3.33)	0.002
Not working	1.51 (1.01, 2.25)	0.043	1.52 (0.98, 2.36)	0.059
**Monthly income**				
≥ $2000	Reference		NA	
< $2000	0.79 (0.35, 1.82)	0.582	NA	NA
**Diabetes**				
Yes	Reference		Reference	
No	1.66 (1.22, 2.26)	0.001	1.80 (1.31, 2.50)	<0.001
**Hypertension**				
Yes	Reference		NA	
No	1.22 (0.82, 1.82)	0.337	NA	NA
**Hyperlipidaemia**				
Yes	Reference		NA	
No	0.99 (0.74, 1.32)	0.928	NA	NA
**Comorbidities**				
<2	Reference		NA	
≥2	0.85 (0.63, 1.14)	0.278	NA	NA
**Smoking status**				
Never smoked	Reference		Reference	
Ex-smoker	1.02 (0.67, 1.56)	0.909	1.11 (0.70, 1.75)	0.649
Current smoker	1.85 (1.08, 3.19)	0.025	1.66 (0.93, 2.95)	0.086
**Eyecare utilization pattern**				
No glasses	1.09 (0.81, 1.47)	0.569	NA	
No yearly glasses checks	1.37 (0.85, 2.21)	0.189	NA	NA
**Aware of vision deterioration**				
Yes	Reference		Reference	
No	1.35 (1.01, 1.82)	0.045	1.20 (0.86, 1.66)	0.290
**Ocular characteristics**				
Laterality of visual impairment				
Unilateral visual impairment	Reference		Reference	
Bilateral visual impairment	2.05 (1.52, 2.79)	<0.001	2.56 (1.81, 3.63)	<0.001
Visual functioning score	1.13 (1.02, 1.24)	0.015	1.26 (1.12, 1.42)	<0.001

Abbreviations: CI = confidence interval; NA, not applicable; OR = odds ratio.

* Model 1: Adjusted for age, gender and ethnicity.

† Model 2: Adjusted for age, gender, ethnicity, housing type, living arrangement, education, employment, diabetes, smoking, awareness of vision deterioration, laterality of visual impairment, and visual functioning score.

**Fig 2:**
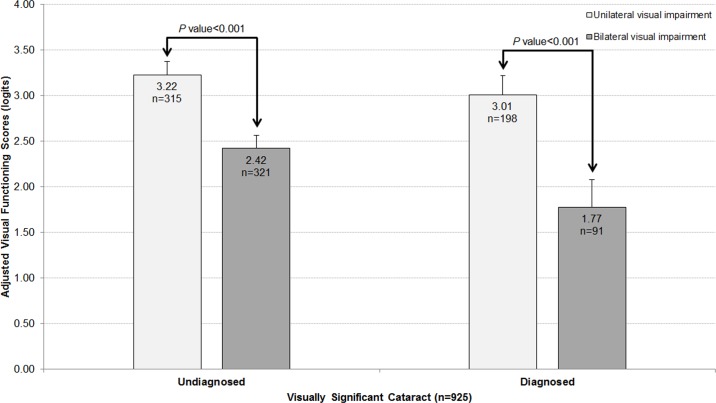
Distribution of the visual functioning scores of participants with undiagnosed vs. diagnosed visually significant cataract stratified by laterality of visual impairment. 50.5% (n = 321) of the undiagnosed participants suffered from bilateral visual impairment, which resulted in a significantly lower visual functioning score than those with unilateral visual impairment (P<0.001 for both undiagnosed and diagnosed groups). Error bars represent 95% confidence intervals. Data and P values shown are after adjustment for age, gender, ethnicity, housing type, living arrangement, education, employment, diabetes, smoking, and awareness of vision deterioration.

Risk factor analyses were performed for subgroups, consisting of those (a) having unilateral visual impairment (n = 513) and (b) bilateral visual impairment (n = 412). In the subgroup analysis of individuals with unilateral visual impairment, persons with undiagnosed visually significant cataract had similar visual functioning score compared to those with diagnosed visually significant cataract (OR, 1.17, 95%CI, 0.99–1.39; *P* = 0.065; **Fig 2**). A similar subgroup analysis was next conducted on individuals with bilateral visual impairment, and persons with undiagnosed visually significant cataract had better visual functioning score compared to those with diagnosed visually significant cataract (OR, 1.31, 95%CI, 1.10–1.55; *P* = 0.002; **Fig 2**).

### Impact of undiagnosed visually significant cataract on visual functioning

**Fig 2**shows the negative impact of undiagnosed visually significant cataract on visual functioning. For both undiagnosed and diagnosed groups, persons with bilateral visual impairment had a significant decrement in visual functioning score compared to those with unilateral visual impairment (*P*<0.001 in both groups; **Fig 2**).

## Discussion

In this multi-ethnic, population-based study of adult Singaporeans, we found that (1) two-third of individuals with visually significant cataract were previously undiagnosed; (2) independent factors associated with undiagnosed visually significant cataract were Malay ethnicity, lower educational attainment, employment, and without a history of diabetes; (3) approximately half of the undiagnosed participants had bilateral visual impairment, which was significantly associated with poorer visual functioning compared to unilateral visual impairment. Our research suggests that cataract remains undiagnosed in many Asian individuals and has a substantial negative effect on visual functioning once vision in both eyes is compromised.

Few studies have explored undiagnosed visually significant cataract in population-based samples. Varma and colleagues reported a prevalence of ~60% of undiagnosed visually significant cataract among Hispanic persons living in the United States, which was similar to that (~70%) in the current study.[28] Such a high rate of undiagnosed cataract in economically developed countries is unexpected, given that eye care services are easily accessible and partially or fully subsidized. Moreover, the cataract surgery rate in Singapore (356 cataract operations per 100,000 persons per year) meet and even exceed that of several economically developed nations.[29] Lack of awareness of visually significant cataract could be due to many reasons. First, cataract develops progressively over many years, is painless, and affects both eyes, which makes it difficult for older persons to notice such gradual changes. As seen in our study, individuals with unilateral impairment tend to seek help earlier because they may have the opportunity to compare the vision in both eyes and “notice” the disparity between eyes than those with bilateral impairment. Among those that unaware of their visual deterioration, visually significant cataract would often only be diagnosed fortuitously. Next, even among persons aware of visual deterioration, not all may have sought ophthalmological assessment in a timely manner. The reasons for not seeking early ophthalmological assessment could be manifold. On a patient level, reasons could include poor knowledge, thus tending to attribute visual deterioration to a normal aging process. Cultural factors may also contribute, as Asians may be more stoic about their symptoms. Studies examining mental health have proposed that Asians have higher symptom thresholds before seeking medical attention, as compared to their Caucasian counterparts.[30] Therefore, it is possible that Asians may have a greater tolerance to blurred vision. This suggests room for improvement in eye health awareness, especially with regards to ensuring elderly patients and their family members are made more aware of new symptoms or deteriorating vision and should not hesitate to seek an ophthalmic assessment.

Disparities by ethnicity, educational and employment status are known to be related to the use of eye care services.[31] We found that rates of undiagnosed visually significant cataract were significantly higher among Malays than any other ethnic group studied in Singapore. We had previously reported a higher proportion of Malays[32] compared to Chinese or Indians having undiagnosed eye diseases such as glaucoma[33] diabetic retinopathy,[34] and vision-threatening retinopathy.[34] This may be related to the known tendency of Malay/Muslims to take a more passive approach to illness.[35] Apart from ethnicity differences, we found that employed persons were more likely to have undiagnosed cataract than retired individuals. We postulate that Asian individuals may be reluctant to seek eye care services, even for symptoms of visual loss, due to difficulties taking time off work. A similar finding from India reported adults who were employed were less likely to seek cataract surgery than those who were unemployed.[36] Employers and workers should be targeted for public health education on symptom awareness and the importance of seeking regular eye examination.

We found that persons with a history of diabetes were more likely to have diagnosed visually significant cataract than those without diabetes. In Singapore, patients with diabetes attend an annual diabetic retinopathy screening program in community polyclinics.[37] The screening process with digital retinal photographs may inadvertently detect cataract when the photographs do not meet sufficient quality standards for interpretation. This is in contrast with a study from United States where persons with diabetes were more likely to have undetected eye disease compared to those without diabetes.[28] However, in this study, the different types of eye diseases were not separately reported making it difficult to draw comparisons with our study. Thus, the provision of subsidized regular vision screening within community polyclinics perhaps may have enabled easier access to and consequently more use of eye care.

This is the first population-based Asian study demonstrating a negative impact of undiagnosed visually significant cataract on visual functioning. Individuals with visually significant cataract had significant deficits in visual functioning, which represent more difficulty in visual tasks, once vision in both eyes is compromised. Our data suggest that those undiagnosed individuals with bilateral visual impairment (approx. 50%) may greatly benefit from knowing their cataract status, which can then lead to possible cataract surgery and an improvement in their visual functioning.[38] Identification of individuals with cataract affecting only one eye is important, as visual functioning is relatively preserved, and referral to ophthalmologist for disease monitoring is effective in preventing progression to bilateral visual impairment when visual functioning is compromised. These findings may serve as additional clinical considerations when determining whether some form of vision screening for impaired visual acuity, or vision impairment, among older adults is worthwhile.[39, 40]

Strengths of this study include its population-based nature, standardized cataract cutoffs to those used by the Eye Diseases Prevalence Research,[41] large sample size (8,697 residents), ethnic diversity (Chinese, Malay and Indian ethnic origin), good participation rates (at least 75%), and use of Rasch analysis to provide interval level scoring for our visual functioning questionnaire. Our study, however, has a number of limitations. First, the knowledge of any prior physician diagnosis of cataract was obtained through self-report, which can be subject to recall bias. Although trained interviewers ensured consistency in asking questions during the face-to-face interview, self-reported ocular history data without validation from medical records could have reduced reliability and accuracy. Second, differential access to eye care services is mitigated to some extent in Singapore, where all citizens and permanent residents are covered by Medisave.[42] Third, perceptions of vision loss are greatly influenced by cultural factors; therefore findings may not be broadly generalizable to other populations outside of Singapore. However, these findings still have direct relevance to other groups of Chinese, Indian or Malay ethnicity living in urbanized societies. Finally, we did not collect data on cost, transport, lack of time, unawareness/lack of knowledge about cataract, beliefs, and attitudes to health, so we do not fully understand *why* people are not accessing eye care services for cataract diagnosis. Future studies using quantitative and qualitative methodology are needed to explore the specific reasons for not seeking medical advice in order to inform interventions at the community and public policy level.

## Conclusions

In conclusion, we found that two-thirds of Singaporean adults with visually significant cataract were undiagnosed. Half of which were affected by bilateral visual impairment, resulting in a significant functional decline. These data highlight the importance of public health strategies targeting elderly patients, such as regular screening for visual impairment and timely referral to ophthalmologists.

## Supporting Information

S1 Table Characteristics of Participants Included and Excluded from the Analysis. (DOCX)
