# Supplementary material for: Prevalence, Risk Factors, and Impact of Undiagnosed Visually Significant Cataract: The Singapore Epidemiology of Eye Diseases Study
Source: PLoS One. 2017 Jan 27;12(1):e0170804. doi: 10.1371/journal.pone.0170804 (PMC5271362; doi:10.1371/journal.pone.0170804)
Supplement: S1 Table — (DOCX) [file pone.0170804.s001.docx]

**S1 Table. Characteristics of Participants Included and Excluded from the Analysis**

| **Characteristics** | **Included** | **Excluded** | ***P* value *** |
| --- | --- | --- | --- |
|  | **(n = 8697)** | **(n = 609)** |  |
| Age, years | 57.74 (9.91) | 60.56 (10.06) | <0.001 |
| **Gender** |  |  |  |
| Male | 4286 (49.28) | 305 (50.08) | 0.702 |
| Female | 4411 (50.72) | 304 (49.92) |  |
| **Ethnicity** |  |  |  |
| Chinese | 2921 (33.59) | 165 (27.09) | <0.001 |
| Malay | 2944 (33.85) | 181 (29.72) |  |
| Indian | 2832 (32.56) | 263 (43.19) |  |
| **Housing type** |  |  |  |
| Big | 8077 (92.98) | 543 (90.05) | 0.007 |
| Small | 610 (7.02) | 60 (9.95) |  |
| **Living arrangement** |  |  |  |
| Living with others | 8267 (95.25) | 567 (94.19) | 0.237 |
| Living alone | 412 (4.75) | 35 (5.81) |  |
| **Marital status** |  |  |  |
| Married | 6820 (78.50) | 469 (77.91) | 0.733 |
| Never married, separated, divorced, widowed | 1868 (21.50) | 133 (22.09) |  |
| **Education** |  |  |  |
| > 6 years | 3565 (41.02) | 228 (37.81) | 0.121 |
| ≤ 6 years | 5125 (58.98) | 375 (62.19) |  |
| **Employment status** |  |  |  |
| Employed | 4870 (56.00) | 293 (48.11) | <0.001 |
| Retired | 1328 (15.27) | 118 (19.38) |  |
| Not working | 2499 (28.73) | 198 (32.51) |  |
| **Monthly income** |  |  |  |
| > $2000 | 2029 (23.83) | 105 (17.98) | 0.001 |
| < $2000 | 6486 (76.17) | 479 (82.02) |  |
| **Diabetes** |  |  |  |
| No | 6475 (77.52) | 370 (66.07) | <0.001 |
| Yes | 1878 (22.48) | 190 (33.93) |  |
| **Hypertension** |  |  |  |
| No | 3530 (40.70) | 198 (32.73) | <0.001 |
| Yes | 5143 (59.30) | 407 (67.27) |  |
| **Hyperlipidaemia** |  |  |  |
| No | 4745 (56.22) | 288 (50.70) | 0.001 |
| Yes | 3695 (43.78) | 280 (49.30) |  |
| **Comorbidities** |  |  |  |
| <2 | 5411 (64.85) | 306 (55.04) | <0.001 |
| >2 | 2933 (35.15) | 250 (44.96) |  |
| **Smoking status** |  |  |  |
| Never smoked | 6016 (69.24) | 420 (70.00) | 0.224 |
| Ex-smoker | 1221 (14.05) | 94 (15.67) |  |
| Current smoker | 1452 (16.71) | 86 (14.33) |  |

Data presented are mean (standard deviation) or number (%), as appropriate.

*P value was obtained with Kruskal-Wallis for the non-normally distributed continuous variables and with chi-square tests for categorical variables.
